# Recycling and Sustainability of Cement-Based Materials: Properties, Applications and Challenges

**DOI:** 10.3390/ma19071281

**Published:** 2026-03-24

**Authors:** Yuanzhan Wang, Linjian Wu

**Affiliations:** 1 State Key Laboratory of Hydraulic Engineering Intelligent Construction and Operation, Tianjin University, Jinnan District, 135 Yaguan Road, Tianjin 300072, China; 2 National Engineering Research Center for Inland Waterway Regulation, School of River and Ocean Engineering, Chongqing Jiaotong University, Chongqing 400074, China; wljabgf@126.com

The extensive application of cement-based composite materials in the construction industry has consumed large quantities of natural resources and energy, causing serious ecological and environmental problems [1,2]. Utilizing industrial waste products to develop recycled and sustainable cement-based materials has become a global research hotspot [3,4]. Research on the development and application of green cement-based materials [5,6], the recycling and performance enhancement of waste cement-based materials [7,8], and the durability of reinforced concrete structures using waste cement-based materials [9,10] is vital for achieving sustainable development. This Special Issue (SI), “Recycling and Sustainability of Cement-Based Materials: Properties, Applications and Challenges”, presents recent theoretical, experimental, and model research findings regarding the properties, applications, and challenges for the recycling and sustainability of cement-based materials. This Editorial summarizes the eight publications (seven research articles and one review article) included in this SI.

Compared with conventional mineral admixture concretes produced by partially replacing cement with industrial waste products, high-performance concrete (HPC), and ultra-high-performance concrete (UHPC) have attracted increasing attention due to their green and low-carbon attributes, as well as superior mechanical properties and durability [11,12], and have been widely used in practical engineering. While maintaining these performance advantages, further reducing material costs and enhancing the degrees of greenness and resource reuse are important research topics in the fields of HPC and UHPC [13,14]. Manganese slag (MS) is an industrial waste produced during metallurgy and chemical industry [15,16], and its application in HPC and UHPC remains to be explored. Xu et al. [17] reported that the incorporation of 0~40% MS could effectively improve the densification and early mechanical properties of UHPC and achieve the solidification of toxic substances, providing a novel technical route for the resource utilization and solidification of MS. Yang et al. [18] further stated that the volcanic ash effect of MS contributed to the refinement of cementitious matrix, and the bridging effect of Basalt Fibers (BFs) could inhibit crack propagation. The synergistic interaction between both significantly enhanced the resistance of HPC to salt erosion.

Fibrous materials can enhance concrete properties by improving inter-component cohesion and promoting the formation of additional calcium silicate hydrate gels through hydration [19,20]. Nanoparticles, with their unique size effects and high surface activity, contribute to the refinement of pore structures [21,22]. BFs derived from natural basalt are regarded as a relatively environment-friendly reinforcing material [23]. Calcium sulfate whiskers (CSWs) can be produced from waste gypsum, exhibiting considerable potential for resource utilization [24]. Zhang et al. [25] demonstrated that the addition of CSWs and BFs could enhance the compressive strength and dynamic elastic modulus of concrete primarily by reducing the proportion of large and capillary pores while increasing the fraction of transition pores. This effect would be further enhanced when CSHs were modified with sodium hexametaphosphate.

Construction and demolition waste (CDW) contains approximately 70% concrete and brick waste, which can be used as recycled coarse aggregate (RCA) to replace natural coarse aggregate (NCA) to produce recycled aggregate concrete (RAC), thus reducing environmental impact, conserving natural resources and decreasing material costs [26,27,28]. The variability in the composition and physical properties of CDW may cause different property defects in RCA, necessitating adaptive strategies to meet the design standards of strength and impermeability. Banyai et al. [29] compared the mechanical properties of RAC produced from different construction demolition sources, including reinforced pool, precast products, pure concrete demolition, and mixed demolition waste. Results showed that by optimizing the water–binder ratio and using special additives such as superplasticizers, RAC could meet the standard requirements for compressive strength and water resistance exposure classes despite RCA not meeting the required standards.

To overcome the inherent limitations of RCA, such as high porosity, large water absorption, and relatively low mechanical properties, various pre-treatment and enhancement strategies have been proposed [30,31,32]. Among them, the carbonation treatment of RCA with carbon dioxide can not only improve the properties of RCA but also achieve the fixation of carbon dioxide, which has attracted extensive research attention in recent years [33,34,35]. The inherent properties of RCA and the original concrete’s performance play a key role in the carbonation efficiency and RAC properties. Ju et al. [36] found that a completely dried initial moisture condition was more favorable for the carbonation of RCA. In addition, RCA derived from the original concrete’s either excessively low or high strength exhibited the reduced carbonation efficiency, attributed to the insufficient carbonatable phases in low-strength concrete and limited carbon dioxide penetration in high-strength concrete. Qin et al. [37] revealed that for RCA derived from low-strength concrete, larger aggregate particles exhibited the more pronounced carbonation enhancement, whereas for RCA originating from high-strength concrete, smaller aggregate sizes demonstrated the higher carbonation efficiency. Li et al. [38] stated that under complete drying condition, carbonated RCA with a particle size of 10~20 mm showed improvement in the optimal physical properties. The workability, compressive strength, and chloride penetration resistance of carbonated RAC prepared under this condition were significantly better than those of ordinary RAC.

Numerous studies have been conducted on the mechanical properties, chloride transport characteristics, and durability of RAC [39,40]. In recent years, enhancing the chloride penetration resistance and corresponding durability of RAC has become a critical research topic for the application and maintenance of reinforced concrete structures using RAC [41,42]. Wang et al. [43] systematically reviewed the enhancement methods of properties, the effectiveness of current methods in improving chloride penetration resistance, the key factors influencing chloride transport characteristics, and serving life prediction and related models of RAC, providing a reference for the application and durability evaluation of RAC.

Overall, research on recycled and sustainable cement-based materials will continue to advance rapidly. On the one hand, future studies should pay more attention to in-depth investigations into existing recycled and sustainable materials. Promising research directions include the adoption of multi-component blending strategies involving different industrial waste products to fully exploit their synergistic effects and overcome the performance limitations using only the waste material [44,45]; the use of long-term natural diffusion tests and in-site service monitoring to investigate the time-dependent performance evolution of recycled and sustainable cement-based materials under complex aggressive environments [46,47]; the investigation of key durability issues of reinforced concrete structures using polynary recycled and sustainable cement-based materials, such as chloride, sulfate transport characteristics [48,49], and chloride threshold content [50]; and the establishment of a comprehensive performance evaluation system of cement-based materials mixed with various industrial wastes (properties, resources, energy, environment, economy, etc.) from a full life-cycle perspective [51]. On the other hand, future research can focus on the development of new recycled and sustainable materials, including recycled and sustainable materials with low cost, superior performance, and strong adaptability, as well as materials with green, intelligent, self-detection, and self-healing characteristics, among others, alongside related property studies. Such efforts are of great significance for the sustainable development of cement-based materials.
